# Advances in the Diagnosis of Venous Thromboembolism: A Literature Review

**DOI:** 10.3390/diagnostics10060365

**Published:** 2020-06-02

**Authors:** Harish Patel, Haozhe Sun, Ali N. Hussain, Trupti Vakde

**Affiliations:** 1 Department of Medicine, BronxCare Hospital Center a Clinical Affiliate of Mt Sinai Health Systems and Academic affiliate of Icahn School of Medicine, Bronx, NY 10457, USA; HSun@bronxleb.org (H.S.); alinhussain13@gmail.com (A.N.H.); TVAKDE@bronxleb.org (T.V.); 2 Division of the Pulmonary and Critical Care, BronxCare Hospital Center a Clinical Affiliate of Mt Sinai Health Systems and Academic Affiliate of Icahn School of Medicine, Bronx, NY 10457, USA

**Keywords:** VTE diagnosis, review VTE, pregnancy and VTE

## Abstract

The incidence of venous thromboembolism (VTE), including lower extremity deep vein thrombosis (DVT) and pulmonary embolism (PE) is increasing. The increase in suspicion for VTE has lowered the threshold for performing imaging studies to confirm diagnosis of VTE. However, only 20% of suspected cases have a confirmed diagnosis of VTE. Development of pulmonary embolism rule-out criteria (PERC) and update in pre-test probability have changed the paradigm of ruling-out patient with low index of suspicion. The D-dimer test in conjunction to the pre-test probability has been utilized in VTE diagnosis. The age appropriate D-dimer cutoff and inclusion of YEARS algorithm (signs of the DVT, hemoptysis and whether PE is the likely diagnosis) for the D-dimer cutoff have been recent updates in the evaluation of suspected PE. Multi-detector computed tomography pulmonary angiography (CTPA) and compression ultrasound (CUS) are the preferred imaging modality to diagnose PE and DVT respectively. The VTE diagnostic algorithm do differ in pregnant individuals. The prerequisite of avoiding excessive radiation has recruited planar ventilation-perfusion (V/Q) scan as preferred in pregnant patients to evaluate for PE. The modification of CUS protocol with addition of the Valsalva maneuver should be performed while evaluating DVT in pregnant individual.

## 1. Introduction

Venous Thromboembolism (VTE) includes thrombotic disease of venous system, but primarily includes lower extremity deep vein thrombosis (DVT) and pulmonary embolism (PE). Incidence for first event of acute VTE is 0.7–1.4 per 1000-person year [1,2,3] with DVT being almost twice more common than PE [3]. The exact incidence for the VTE is not known due to lack of national registry. However, it is estimated that annual incidence of VTE is around 300,000–600,000 cases [4].The incidence of the VTE has increased over time, primarily due to increase in diagnosis of PE [5]. The incidence of DVT has plateaued over time [6]. Advances in imaging modality have increased sensitivity for diagnosis of sub-segmental PE and hence leading to increase in incidence of PE [7]. There has been increment in number of hospitalizations due to PE. However, severity and subsequent mortality from PE have declined over time [7,8].

The clinical presentation of DVT comprises of swelling of the lower extremity, calf pain, inflammatory changes like increase in warmth and edema. In the absence of treatment, DVT can progress to PE in 30–50% of individuals [1]. Pulmonary embolism presents with hemoptysis, shortness of breath, pleuritic chest pain and often with hemodynamic instability in the form of hypotension and hypoxia. However, the accuracy of clinical diagnosis of DVT is limited, with only half of the patients with DVT have presenting symptoms or positive findings on pertinent physical exam [9]. The presentations of cellulitis, hematoma, venous phlebitis may often confound the diagnosis of DVT. The clinical manifestations of the PE are similar to acute coronary syndrome, congestive heart failure, varied pulmonary hypoxic manifestation, hemodynamic instability due to sepsis. Anti-coagulation, to prevent the thrombus progression, remains a main stay to the management of patients with hemodynamic stable VTE and it is prudent to establish diagnosis before concluding to long-term anti-coagulation therapy. Imaging modality are required to establish the diagnosis of the VTE.

It is estimated that only 20% of suspected cases of VTE have a confirmed diagnosis with imaging studies [10]. Clinical algorithms are utilized in reallocating the imaging resources for high index cases. Clinical decision rules triage patients with suspected DVT and PE who will benefit for the VTE directed imaging studies. Utilization of the clinical parameters from Wells score for suspected DVT and revised Geneva score for PE categorizes the clinical presentation as, VTE likely or VTE unlikely. Clinical decision scoring in conjunction with serum D-dimer test assist in ruling out VTE in patients with low suspicion of VTE. This systemic diagnostic approach using pre-test probability and D-dimer testing assist in excluding DVT or PE in an estimated 28% of patients [11,12].

Pregnancy is a pro-coagulant state [13]. In conjunction to the pregnancy-induced thrombophilia, venous stasis from the gravid uterus poses a fourfold increase in the incidence of the DVT during pregnancy [14]. Pregnancy related lower extremity edema cofounds the clinical presentation of DVT. There is a physiological elevation of the D-dimer during pregnancy, hence it’s utility is limited in pregnancy to rule out DVT. The diagnostic algorithm for PE during pregnancy accounts to prevent excessive radiation exposure to pregnant patients. Hence, the diagnostic approach of the VTE during pregnancy is distinct from non-pregnant individuals.

It is well established that malignancy with or without chemotherapy is a risk factor for developing VTE. Approximately 20% of unselected VTE cases are associated with active cancer [15]. Depending on the type of cancer and the anti-neoplastic regimen, this risk may further increase [16]. The utility of the pre-test clinical probability and the D-dimer for initial VTE evaluation in patient with cancer is not clear and they should directly proceed to diagnostic imaging studies [17].

The review article discusses the advances in the diagnosis of the VTE. The recent updates in the clinical algorithms for excluding the VTE and avoiding the unnecessary testing, evaluating the probability for the VTE, D-dimer for the ruling out VTE, the advances in the imaging studies for VTE evaluation and approach in distinct clinic condition like pregnancy and malignancy. The diagnostic approach differs in patients with prior malignancy and has been discussed in the concluding section of the article.

## 2. Diagnosis of the Pulmonary Embolism in a Non-Pregnant Individual

Thromboembolic disease is a leading cause of mortality worldwide, accounting for 1 in 4 deaths and an estimated 34% of patients die within few hours of presentation [18,19]. There is increasing awareness for PE amongst health care professional and with advancement in the non-invasive testing, predominantly with the computerized pulmonary angiogram (CTPA) health care professional initiate the testing for the PE more frequently. Only 5% of the individuals have a confirmation of PE from the diagnostic study as compared to 50% in the past [20]. It is critical to assess clinical criteria with high diagnostic yield for PE and construct a decision rule for evaluating the need for the imaging studies. The majority of the recent advances in evaluation of the PE have contributed to ruling out low probability cases prior to diagnostic imaging studies. In this section we include the discussion of these advances, that is comprised of the new clinical decision rule, advent in the age appropriate d-dimer cutoff and modification of the Geneva score of assessing the probability of PE. In conjunction to diagnosis of PE it is imperative to assess for the severity and risk of early mortality.

### 2.1. Clinical Presentation

Predisposing risk factors for PE often accounted for the diagnostic work-up, however, 40% of patient with acute PE are devoid of risk factors [21].The clinical presentation of acute PE is non-specific and at times asymptomatic with an incidental diagnosis on imaging studies. Acute PE is suspected in patients with dyspnea, chest pain and syncope [22]. The presentation with syncope often has hemodynamic instability and incurs worse outcomes due to right ventricular strain [23].

### 2.2. Pulmonary Embolism Rule-Out Criteria (PERC)

The emergency room clinical assessment, driven by PERC, identifies the patient with the low likely hood of pulmonary embolism and further diagnostic work-up can be deferred. It comprises of eight clinical criteria (Box 1). These criteria were calculated from retrospective logistic regression analysis of 21 clinical variable derived from 3148 patients in United States emergency department [24]. The PERC rule was applied on low and very-low risk patients with an estimated prevalence of 8% and 2% respectively. PERC did not comprise of the D-dimer testing. The clinical application of PERC in low and very-low risk individuals had low specificity of 27% and 15% and an impressive sensitivity of 96% and 100% to diagnose PE in non-selected patients [24].The prospective observational study in Europe have validated PERC in low risk emergency department patients [25,26]. The results from PROPER trial, a randomized control study in European population, showed that PERC was non-inferior to the conventional evaluation of PE [26]. The Study showed that patient randomized to the PERC arm were diagnosed with PE in 1.5% vs 2.7% in conventional group and difference of the patients undergoing CTPA was much lower in PERC group (13%) as compared to conventional group (23%). It is important to note that PERC is validated in the low risk individuals only and carries a limited applicability in high-risk group, like malignancy. PERC is validated in emergency department with low prevalence of PE; there is lack of data to support generalization.

Box 1 **Pulmonary Embolism Rule-Out Criteria [24].**

(i) Age of 50 years and above
(ii) Pulse rate of at-least 100 beats per minute
(iii) Pulse oximetry with oxygen saturation below 95%
(iv) Unilateral leg swelling
(v) Hemoptysis
(vi) Recent surgery or trauma
(vii) Prior history VTE (DVT or PE)
(viii) Oral hormone use

### 2.3. Assessment of Pre-Test Probability

Assessment of the predisposing factors and clinical signs or symptoms compute the pre-test probability. Empiric clinical judgement or established clinical decision rule can be implemented to evaluate the pre-test probability of PE. Physician can practice empiric clinical judgement to assess the pre-test probability by assessing patient’s symptoms and prevalent diagnostic test like chest x-ray or ECG. Empiric clinical judgement has been tested in several settings, however, it lacks the standardization [27]. The two most prevalent standardized clinical decision rule are the Wells Rule and the Geneva score. Wells score in conjunction to the D-dimer test has been validated in several clinical trials [28]. Both clinical decision rules have limitation to its clinical implementation. However, revised Geneva score is a fully standardized clinical decision rule, independent of the clinical judgement and hence reproducible. The clinical implementation of revised Geneva score has been compared to Wells rule for calculating the pre-test probability for PE in randomized clinical trials [29]. Meta-analysis from 11 key studies comparing two prediction rule revealed that Wells score is more accurate in diagnosing PE in hospitalized elderly, with area under the receiver operating characteristic curve of 0.91 (95% confidence interval (cl), 0.85–0.98) for the Wells score as compared to 0.69 (95% cl, 0.56-0.82) for the revised Geneva score [30]. As per current recommendation either of the scoring, Wells or revised Geneva, system can be used. Some experts due to reproducibility prefer revised Geneva system and it overcomes the inter-observer variability. The clinical probability for PE as per revised Geneva score can be a three-level (low, intermediate or high probability) or two level score (PE-likely or PE-Unlikely) [31] (Table 1). Based on this prediction rule around 12% of the patients with ‘PE-unlikely’ and 30% of patient with “PE-likely” are expected to have confirmed PE [32].

### 2.4. D-Dimer

Acute thrombosis increases D-dimer levels and it is a sensitive marker of thrombosis generally. The high negative predictive value of D-dimer is utilized in the clinical conditions to rule out PE. The d-dimer levels increase with age, and in other clinical conditions like cancer, pregnancy, inflammatory states; Hence, limits its positive predictive values for diagnosis of PE [33].

D-dimer testing is indicated in patients who have low or intermediate probability of PE. Physiological D-dimer levels do vary with age and hence age adjusted cut-offs for the D-dimer have been studied to reduce false positive rate in PE. The D-dimer cut-off in individuals older than 50 years of age is calculated by multiplying as patient age by 10 ng/dL [34]. ADJUST-PE study, a multinational trial with 3346 patients evaluated the need of the CTPA based on the age adjusted D-dimer. Study protocol included 3-month follow-up for the patients with normal age adjusted D-dimer. There were 766 patient above 75 years with non-high clinical probability and application of the age adjusted D-dimer excluded 200 (30%) of the individuals as compared to 46 (6.4%) with the conventional D-dimer cutoff [34]. This approach of the age adjusted D-dimer testing in patients with low probability (PE-unlikely) has a 5% increase in the proportion of patient in whom the imaging studies can be withheld [12].

The pre-test probability-based D-dimer cut-offs have been proposed using YEARS decision rule [35]. This multi-center European trial evaluated patient for the YEARS criteria (signs of the DVT, hemoptysis and whether PE is the likely diagnosis). If no criteria were met, the D-dimer cut-off was 1000 ng/dL and if one or more criteria was present, then cut-off was considered 500 ng/dL. As oppose to ADJUST-PE protocol YEARS criteria has refined D-dimer cut-off based on clinical probability. The study evaluated cohort of the 3465 patients with YEARS algorithm with a 3-month follow-up. There were 18 patients diagnosed with VTE during 3-month follow-up. CTPA was not performed in 48% of patient with YEARS criteria, if the Wells score algorithm was applied, then only 34% of patients would have been excluded. YEARS algorithm decreased the need for imaging studies by 14%. The YEARS protocol it yet to be tested in other clinical trials.

D-dimer testing is performed using several commercially available assays. The sensitivity and specificity of the D-dimer level vary for different assays. Attempts to standardize D-dimer assay have not been successful [36]. There have been recent advances in the point-of-care D-dimer testing, specifically for remote areas [37]. A systemic meta-analysis revealed the sensitivity of the point of care testing for the D-dimer to be 88% as compared to the conventional D-dimer testing of 95% [38]. Hence, extra-caution should be executed in utilizing the point of care D-dimer to rule out PE.

Patients with high pre-test probability for the PE (PE-likely) should not be tested for D-dimer. D-dimer has low specificity and hence cannot be used to rule in a diagnosis of PE [39]. It is recommended that patient D-dimer levels will not change recommendation for imaging studies in patients with a high probability of PE. D-dimer should be used in clinical presentation where the pre-test probability of PE is low.

### 2.5. Biomarker for VTE

D-dimer is a key biomarker implemented in the clinical practice. There have been advents in development of new biomarker, few in conjunction with D-dimer, to increase the specificity of diagnosis of VTE.

Soluble P-selectin (sP-sel) is one of the novel biomarker for VTE and expressed on activated platelet of a thrombus [40,41]. The sP-sel is a soluble form of the transmembrane P-selectin that is secreted in circulation and noted to be elevated in patient with VTE [42,43]. sP-sel is considered an independent risk factor for VTE in cancer patient [44]. Retrospective data has reported the increase in D-dimer, Factor VIII, and F1+2 levels in conjunction to the sP-sel in patient who have VTE [45]. However, sP-sel is not routinely used in clinical practice.

Microparticles (MPs) are the phospholipid cell membrane fragments derived from the platelet, leucocytes and endothelial cells [46,47]. Elevated levels of the MPs have been associated with the inflammatory, thrombotic and vascular disease. Elevated MPs levels are found in mouse model with the experimental VTE [48]. MPs are elevated in cancer patient with VTE and has been utilized in predicting the VTE in patient with known malignancy [49,50]. Indeed, MPs have not been implemented in the routine clinical practice in work-up of patient with suspected VTE. E-selectin and Interleukin-10 are other biomarkers that are linked to VTE. The combination of the D-dimer MPs and Sp-sel levels with patient risk stratification have been studied to evaluate a predictive model for VTE [43]. This model needs further verification before implementing in clinical practice.

In up to 20% of patients, the unprovoked VTE can be recurrent [51]. Familial studies predict that 50% of the patient with the recurrent VTE, could have a hereditary factor contributing to it [52]. Often there are Genetic testing and single nucleotide polymorphisms (SNP) to predict the susceptibility for the recurrent VTE. However, a single SNP or genetic biomarker may not accurately predict the VTE recurrence and hence predictive model have been utilized to identify those at risk for recurrent VTE [53].

### 2.6. Diagnostic Algorithm for Pulmonary Embolism

**Figure 1:**
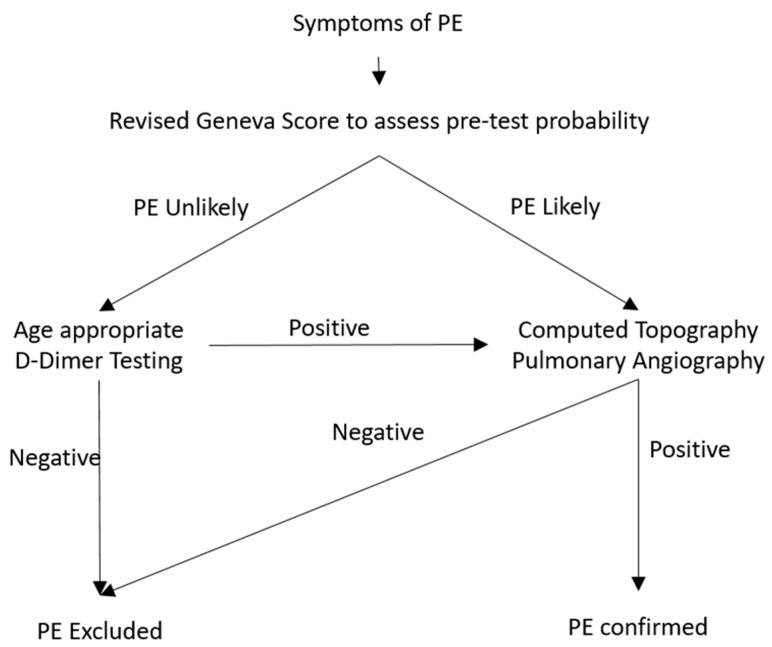
Diagnostic algorithm for suspected pulmonary embolism in a non-pregnant patient.

The algorithm consisting of pre-test probability, D-dimer testing and the Computed tomography pulmonary angiography (CTPA) have been widely accepted [54]. The pre-test probability is assessed as the initial step for the non-high-risk probability of PE and those with high risk should proceed with imaging studies preferably CTPA. The age adjusted D-dimer level evaluates the need of CTPA in the patients with low pre-test probability of PE (Figure 1). The revised Geneva decision score is devoid of variability of inter-personal clinical judgement and reproducible and hence preferred over the Wells Rule evaluating the pre-test probability.

### 2.7. Imaging Modality for Diagnosis of Pulmonary Embolism

Pulmonary angiogram was considered once to be ‘gold standard’ for the diagnosis of PE, but it is invasive and rarely performed due to high risk of complications. Pulmonary angiogram has 0.5% mortality risk, especially for the patients with hemodynamic instability and respiratory failure [55]. CTPA is less invasive, low risk of complication and has an equivalent proficiency comparable to pulmonary angiogram, and is preferred modality for initial evaluation of PE [56]. Pulmonary angiogram has highest radiation exposure of the 10–20 mSv, as compared to 3–10 mSv with CTPA [57].

Multi-detector CTPA is the preferred imaging modality for the diagnosis of PE. The digital subtraction angiography enables visualization of the sub-segmental level of pulmonary artery and has increased the efficacy of CTPA [58]. Pulmonary Embolism Diagnosis (PIOPED) II study evaluated the utility of enhancing the CT pulmonary angiography with a CT venous-phase filling (CTV). Computerized protocol scans the lower extremity after performing pulmonary arterial imaging based on timing of the injection of contrast. The trial showed that the combination of the CTPA and CTV has a higher diagnostic sensitivity and specificity. The diagnostic sensitivity and specificity of CTPA were 83% and 96%, whereas that for the combination of the CTPA-CTV were 90% and 90%, respectively. The pre-test probability does affect the sensitivity and specificity of CTPA. The negative predictive value of CTPA in patients with low or intermediate risk of the PE was 89% to 96%, whereas it is only 60% for the patients with the high risk of PE [59]. There is uncertainty about further investigation in patients with the high-risk pre-test probability and negative CTPA.

Pulmonary perfusion scan is evaluated against the lung ventilation images in planar ventilation-perfusion (V/Q) scan protocol. Lung ventilation scan can be abnormal in individual with lung parenchymal disease, hence x-ray chest should be performed prior to V/Q san. The V/Q scan evaluates for mismatch in the perfusion abnormality due to embolized pulmonary arterial segment and lung ventilation which is expected to be normal in PE. The radioactive tracers like Technetium-99m aersol, Xenon-133 or the Krypton-81 gas are used for ventilation scans. The perfusion study is assessed using Technetium-99 tagged albummacro-aggregate. The findings of the V/Q scan are usually reported through a standardized tier system for an optimal communication [60,61]. The three tier of reporting includes (i) high-probability scan (high probability of PE), (ii) normal scan (PE is rule out) (iii) non-diagnostic scan. Currently there is no literature to opine on diagnostic algorithm utilizing the pre-test probability and V/Q scan for the evaluation of the PE in non-pregnant patients. Though, it is suggested that the high-probability V/Q cannot confirm PE in a patient with low pre-test probability [62]. CTPA is the preferred imaging modality for non-pregnant individual and V/Q scan is preferred for the individual in whom CT scan or intravenous contrast are contra-indicated.

### 2.8. Assessing Severity for Pulmonary Embolism and Diagnostic Approach for Critically Ill Patients with Suspected Pulmonary Embolism

The presentation of hypotension, hypoxia and syncope are manifestations of decreased right ventricular (RV) filling and/or output due to acute RV failure [63]. Echocardiographic finding of RV strain or RV dysfunction is present in 25% of patients with acute PE [64]. The McConnell sign (decreased contractility of RV apex) and ‘60/60’ sign (pulmonary ejection acceleration time with peak systolic tricuspid valve gradient) are suggestive of the PE, but present only in 12%–20% of patient with the PE [65]. This echocardiographic RV finding are not suggestive of the severity, but can be utilized in assessment for hemodynamically unstable patient with suspected PE. Tricuspid annular plane systolic excursion (TAPSE) is decreased in patient with PE and level of less than 16 mm are associated with poor outcome [66]. The increase in RV diameter as compared to the left ventricular volume, denoted by RV/LV index is also indicator of unfavorable outcome. The findings of the RV/LV ratio of >0.9 on CTPA is a predictor of the poor outcome [67].

In patient with high suspicion of PE presenting with the hemodynamic in-stability have a wide array of differentials including congestive cardiac failure, myocardial infarction, aortic dissection, pericardial effusion with cardiac tamponade. Patients are often unstable to be mobilized for CTPA. The echocardiographic RV evaluation can be performed as the initial step. The absence of signs of RV dysfunction should initiate the work-up for alternate etiology. The patient with RV dysfunction should undergo CTPA. In case of in-ability to perform CTPA, anti-coagulation for PE should be considered in patients with RV dysfunction and high probability of PE.

The elevation of troponin levels, marker of the myocardial injury, are associated with poor prognosis of acute PE. Elevated troponin level correlates to mortality in un-selected group of cohort with acute PE and also in hemodynamic unstable patients [68]. The elevated troponin has prognostic implications, however, there is paucity of literature for troponin guided of acute PE. NT-proB-type Natriuretic Peptide (pro-BNP) levels can be elevated in patient with acute PE due to RV myocardial stress. Elevated pro-BNP level from LV etiology is a confounding factor and decreases the specificity of the pro-BNP for assessing outcomes of acute PE. However, owing to its high negative predictive value, the poor outcomes from acute PE can be excluded in patient with normal pro-BNP levels [68].

## 3. Diagnosis of Lower Deep Vein Thrombosis in a Non-Pregnant Individual

The deep vein thrombosis (DVT) is more frequent than PE, carrier a lower morbidity and fatality than PE. There is 2% to 5% mortality for the patient presenting with DVT and no PE [69]. Acute PE is often the sequelae of DVT and likely cause of mortality for patients with acute DVT. The surge in number of clinical evaluations for DVT has a risen necessity for a clinical decision rule to withhold the testing in patients with low likelihood of the DVT. Akin to acute PE, the pre-test probability and the D-dimer play a key role in the initial diagnostic evaluation of DVT.

### 3.1. Pre-Test Probability of First Event of Lower Extremity DVT

Clinical presentation of DVT, like lower extremity swelling, pain, induration of lower extremity, increased warmth lacks acceptable sensitivity and specificity to diagnose DVT. The pre-test probability is calculated through clinical decision rules like Wells Score. The score allocates a two or three tier probability for the DVT (Table 2). The two-level probability for pre-test probability has been preferred for clinical practice [11]. The scoring system is standardized; however, the component of clinical judgement (alternative diagnosis of the DVT) leads to an inter-observer variability. The pre-test probability is only utilized for diagnosis of first event of DVT. The Wells scoring system is not validated for recurrence of DVT.

### 3.2. D-Dimer Level and Diagnostic Algorithm for the Diagnosis of Lower Extremity DVT

**Figure 2:**
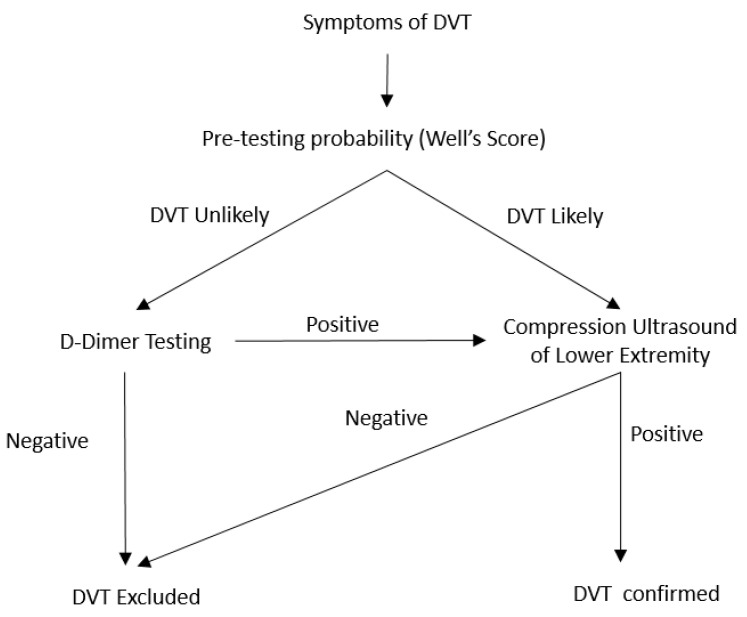
Diagnostic algorithm for the acute DVT in non-pregnant patients.

D-dimer is a product of the cross-linked fibrin noted to be elevated in patient with acute thrombosis and DVT is unlikely in patients with normal D-dimer [70]. Generally, D-dimer level are used in association with the pre-test probability for the diagnostic algorithm for acute DVT. However, non-elevated D-dimer level can exclude the diagnosis of acute DVT in up to 30% of the cases [71]. Currently a single cutoff a value D-dimer value is used to rule out DVT. A meta-analysis looked into 8 studies to evaluate use of age adjusted D-dimer for the excluding DVT. Difference in the DVT prevalence, D-dimer assay and the study design limits clinical utility of the meta-analysis results. However, it showed that by using age-adjusted D-dimer in diagnostic algorithm of DVT the negative predictive value increased from a range of 89.7–100% to 91.8–100% [72]. However, as opposed to acute PE, the use of age adjusted D-dimer has not been adopted in clinical practice. There are ongoing prospective trials for utility of the age adjusted D-dimer in diagnosis of acute DVT (ClinicalTrials.gov: NCT02384135). The D-dimer testing is only done for the patient with the low pre-test probability and those with high-pretest probability for the acute DVT can proceed with imaging studies for the diagnosis (Figure 2).

### 3.3. Imaging Modality for the Diagnosis of Acute DVT

Contrast venography was the conventional ‘Gold Standard’ for diagnosis of acute DVT [73]. Due to patient discomfort, bleeding complications, contrast related adverse event and accessibility issues venography is rarely performed. Inability to feel venous system in 20% of the cases and inter-observer variability are the major technical shortcomings in using venogram in daily practice [74,75]. Contrast venography also poses a risk of thrombosis leading to subsequent DVT [76]. However, venogram is proficient in diagnosis of distal DVT and it can overcome shortcoming of venous ultrasound.

Venous ultrasound (VUS) is the first line and widely accepted imaging modality for the evaluation of DVT [77]. The compression ultrasound (CUS) assess compressibility of proximal lower extremity veins. Inability to compress the lower extremity veins with moderate ultrasound probe pressure diagnoses DVT. The distal vein DVT is evaluated with whole leg US. The VUS carries a sensitivity of 93%for the proximal vein and 63% for the distal vein DVT [78]. The specificity for VUS is 93% for both proximal and distal DVT, but is decreased with application of Doppler with US [78]. VUS is generally performed by the radiology technicians, however emergency room physician can perform ultrasound and expedite evaluation of DVT with compromising the sensitivity or the specificity [79].

Computerized tomographic (CT) venography protocol can be performed for the evaluation of the DVT. The intravenous contrast is injected though peripheral vein without cannulation of the lower extremity and CT images are obtained with timed protocol coinciding with filling of lower extremity vein. CT venography is usually preformed with CTPA in cases where PE is suspected in conjunction with DVT.

Magnetic resonance imaging (MRI) can diagnose DVT, but rarely performed. Visualization of the deep venous structure is improved with IV gadolinium. The venous filling defect is diagnostic of DVT. Alternatively, direct thrombus imaging techniques of MRI can diagnosis DVT without the use of the IV contrast.

## 4. Diagnosis of the PE in Pregnant Individuals

Some pregnant individuals are at increased risk of thrombotic events carry a worse prognosis as compared to non-pregnant counterpart [80]. VTE, comprised of DVT and PE, is one of leading cause of maternal mortality in the US [81]. A prior episode of VTE, pre-eclampsia, post-partum hemorrhage, C-section and still birth are the risk factors of VTE during pregnancy [82]. DVT is more common in ante-partum period, whereas PE is more prevalent in puerperium [83].

The overlapping of common physiological symptoms of pregnancy and acute PE poses a diagnostic challenge. Dyspnea and pleuritic chest pain, like non-pregnant patients, are the most common presenting symptoms for acute PE during pregnancy [84]. However, seventy percent of women have reported dyspnea during pregnancy and hence it is a confounding factor to initiate symptom based evaluation for PE [85]. Rarely acute PE can present with hypotension and shock during pregnancy.

Acute PE is only diagnosed in 2–7% of pregnant patients undergoing testing [86].The utility of D-dimer to exclude PE is limited due to physiological elevation of the D-dimer during pregnancy [87]. D-dimer testing in conjunction with the pre-test probability, CUS and CTPA has revealed positive results but not widely accepted [88]. The study suggested that PE was excluded in 11% of patients based on the normal D-dimer levels.

**Figure 3:**
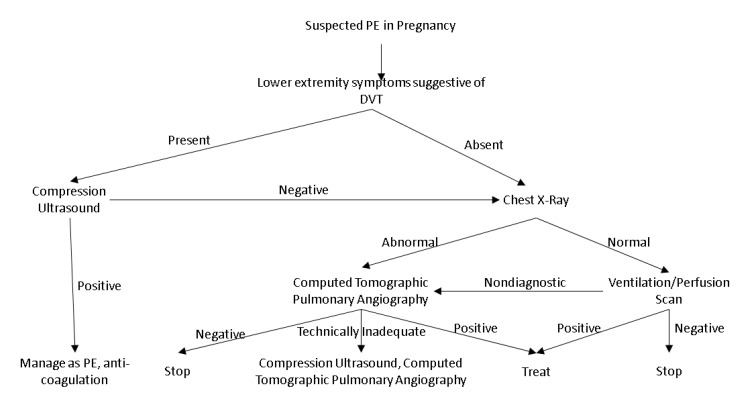
Diagnostic algorithm for evaluation of suspected PE during pregnancy.

The fetal radiation of the more than 50–100 mSv are associated with fetal complication [89]. Planer V/Q has lower radiation exposure than CTPA, 2 mSv and 3–10 mSv respectively. CTPA has been associated with high breast exposure; however, with current advances in the CTPA breast radiation dose is limited to 3–5 mSv [90]. DVT usually precedes acute PE and hence CUS should be performed in patient with lower extremity symptoms [91]. The anti-coagulation therapy can be initiated with presumptive diagnosis of acute PE if CUS is positive in pregnant patients. In those patients with implicit diagnosis of acute PE from positive CUS, clinical acumen and the severity assessment of PE can direct further need of chest imaging to establish the diagnosis (Figure 3). The X-ray of the chest is the first radiological imaging performed in patient with negative CUS. Plan V/Q scan should be performed in patients with normal chest x-ray and those with pulmonary parenchymal abnormality will proceed to CTPA. Magnetic resonance angiography (MRA) requires gadolinium to assess for pulmonary artery feeling defect and the rates of inconclusive results are very high [92]. There is scarcity of data regarding the long-term fetal effect of gadolinium and hence MRA is not a viable option for the evaluation of acute PE during pregnancy.

The use of the thrombolytic therapy raises a concern of high risk of complications. There is an 8% risk of hemorrhage from genital tract [93].Streptokinase and recombinant tissue plasminogen do not cross placenta, yet carries a 6% risk of fetal loss and pre-term delivery [94]. However, as compared to non-pregnant counterpart use of thrombolytic in different clinical condition, including myocardial infarction and stroke, have low complication rate [95]. The catheter directed thrombolytic therapy decreases the thrombolytic dose, however data has been limited to few case reports and its routine use remains unclear [96].

## 5. Diagnosis of the DVT during Pregnancy

DVT is more common in ante-partum period and evenly distributed to all trimester [97]. Venous stasis from gravid uterus in conjunction with pregnancy induced pro-thrombotic state are considered to be likely pathogenesis of the acute DVT. The lower extremity edema is often considered physiological during pregnancy, but may not be related to venous reflux [98]. The majority of the DVT are in the left lower extremity and often associated with ilio-femoral or iliac vein thrombosis [99,100].

The studies for clinical decision rule for DVT have excluded pregnant individuals. Physiological lower extremity swelling without thrombosis limits the evaluation of pre-test probability [101]. In a retrospective accuracy multicenter review, the characteristics of 194 unselected pregnant patients with first suspected DVT were studied. There were 17 patients with confirmed DVT and had at least one of the following three characteristic (i) left lower extremity symptoms, (ii) difference in calf circumference of more than 2 cm and (iii) first trimester of presentation. These three parameters are referred to as the LEFt rule. With a 8.8% prevalence of DVT in this study, the adjusted odds ratio for left lower extremity, difference in calf circumference of > 2 cm and first trimester presentation were 44.3%, 26.9 and 63.4 and with none of the characteristic DVT not diagnosed [100]. However, the LEFt rule is not routinely used in the clinical practice and is utilized in follow-up of patient with negative CUS.

Unlike non-pregnant individuals with lower risk probability, normal D-dimer level cannot rule out DVT in pregnant individual [102]. The serial levels of red blood cell agglutination D-dimer testing (SimpliRED), referred to as highly sensitive D-dimer, has been studied to exclude DVT in pregnancy [103].

The initial evaluation of pregnant patients with suspected DVT should be performed with CUS. Diagnostic algorithm of DVT in non-pregnant patient cannot extrapolated in pregnant patients due to high incidence of pelvic and iliac vein thrombosis [104]. The CUS protocol can be modified by addition of Valsalva maneuver and assessment of venous flow alteration with respiration can increase sensitivity for the iliac vein thrombosis [105]. The CT scan venography has been utilized to diagnose pelvic thrombus, however, there is risk of significant radiation exposure [106]. MR venography in conjunction with the ultrasound has been utilized to diagnose pelvic vein thrombosis [107]. However, routine use of the MR venography is not supported by a prospective trial and it lacks routine availability.

**Figure 4:**
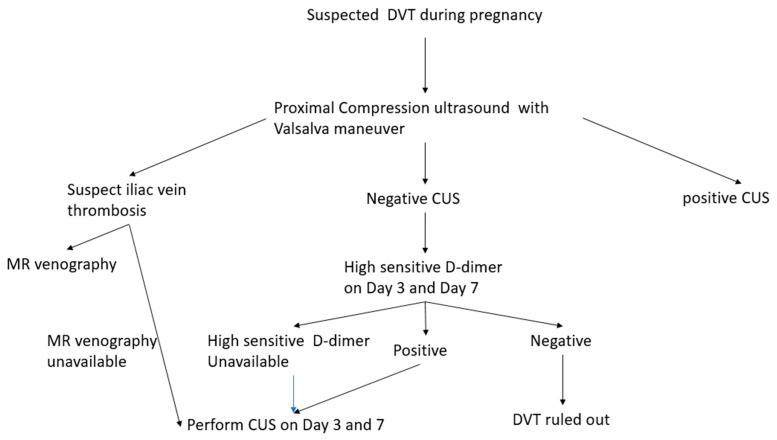
Diagnostic algorithm for evaluation of suspected DVT during pregnancy.

The diagnostic evaluation for DVT in a pregnant individual should begin with CUS (Figure 4). The patients with the negative CUS should be followed on day 3 and day 7. The highly sensitive D-dimer, if available, can performed to decide the need for repeat CUS. If highly sensitive d-Dimer is not available then repeat CUS on day 3 and day 7. The suspected iliac vein thrombosis can be evaluated with MR venography.

## 6. Diagnosis of VTE in-Patient with Malignancy

The malignancy associated hypercoagulable state causing increased risk of cancer-associated thrombosis (CAT) has been well established [108]. Malignancy affects the activities of daily living (ADL) and increases the risk of the thrombosis due to decreased. The increased blood viscosity and cancer related thrombophilia is also increasing the likely hood of CAT [109]. VTE is the second most common cause of the death, after malignancy, in patients with cancer [110]. Thromboprophylasis is the key to decrease the VTE related deaths in patients with underlying malignancy. However, caner associated VTE risk evaluation models are comprehensive and a thorough discussion is beyond the scope of this article.

### 6.1. Limitation of the Pre-Test Probability and D-Dimer in Assessing VTE in Patients with Malignancy

Management of CAT with the anti-thrombotic carries a high risk of the bleeding. Hence, it is imperative to establish that diagnosis prior to the therapeutic anti-coagulation. The pretest probability assessment have been validated in unselected patient cohort, but the utility in the cancer specific group has not been well proven [32]. However, there are less than 20% of cancer patients in the studies done to evaluate the clinical decision rule of PE [111]. The prevalent scoring algorithm like Wells score and revised Geneva score has allocated only one cancer related factor in predicting VTE and it may not suffice the thorough pre-test probability evaluation in patient with malignancy. In a multicenter study, evaluating 3306 patient with suspected PE had 475 patients with cancer, found that Wells rule was less diagnostic for PE in patient with malignancy [112]. It is also important to note that many of the studies have only a small proportion of cancer patients in low risk probability and hence it limits the reliability of the conventional diagnostic approach in patient with cancer. In a pooled analysis of three diagnostic studies of 200 patients from a total cohort of 2696 only 15% (n = 31) had been triaged to low probability as compared to 42% (n = 1049) in patients with no cancer.

The current literature does not support the utility of the D-dimer and pretest probability while assessing the need for imaging studies in cancer associated VTE [17]. It is proposed that patient with cancer suspected to have VTE should have radiology studies for confirmation of PE or DVT [17,113].

### 6.2. Evaluating the Risk of the VTE in Patient with Malignancy

Thromboprophylaxis may be offered to selected high risk outpatients with cancer [114]. Weighing the high bleeding risk, it is utmost important to identify those cancer patients who have high probability of VTE. Khorana et al developed a prediction model using total of five clinical (tumor entity and body mass index) and laboratory (hemoglobin level and thrombocyte and leukocyte count) variables to risk stratify ambulatory cancer patients undergoing chemotherapy into low, intermediate and high risk category [115]. ]. A cut-off of > 3 was initially proposed to identify high risk patients but due to low sensitivity for certain tumors and increasing number of patients falling into intermediate category, more recently a score of > 2 is used to categorize patients as high risk for VTE [116]. This prediction model was expanded further by the Vienna Cancer and Thrombosis Study (CATS) group by adding two new laboratory parameters namely D-Dimer (with a cut-off of 1.44 g/mL) and sP-selectin (with a cut-off of 53.1 ng/mL) and the PROTECT score by adding chemotherapy such as platinum-based regimens and gemcitabine to improve the predictive value [117]. Most recent risk assessment model was developed by the clinical Perceptions and Awareness in real life patients–Cancer Associated Thrombosis (COMPASS–CAT) study which incorporated other variables such as recent hospitalization (<3 months), stage of cancer, anti-hormonal therapy for women with breast cancer or anthracycline-containing chemotherapy, presence of central venous catheter, time since cancer diagnosis, platelet count and personal history of VTE. This tool can be used in patients with common solid cancer receiving anticancer therapy [118].

## 7. Conclusions

VTE is a common clinical condition and there is a surge in the diagnostic testing for PE and DVT. There have been advances in ruling out the low index cases for VTE, based on clinical probability and/or D-dimer testing, prior to the performing the imaging studies. The CTPA and CUS remain the key diagnostic modalities for evaluating PE and DVT, respectively. The management algorithm in pregnant patients are modified for the diagnosis of DVT and/or PE as compared to the non-pregnant individuals. Patients with underlying malignancy and suspected VTE may proceed directly with diagnostic imaging studies.

## Figures and Tables

**Table 1:** Revised Geneva score to assess pre-test probability of PE [31].

Items	Rule Points
Previous PE or DVT	1
Heart Rate	
75-94 BPM	1
≥95 BPM	2
Previous Surgery or Fracture	1
Hemoptysis	1
Active Cancer	1
Unilateral Leg Pain	1
Pain on lower limb palpation and unilateral edema	1
Age >65 Years	1
Clinical Probability	
Three-point score	
Low	0–1
Intermediate	2–4
High	≥5
Two-point Score	
PE Unlikely	0–2
PE Likely	≥3

**Table 2:** Well score (clinical decision rule for DVT).

Clinical Variable	Points
Active Cancer	+1
Paralysis, Paresis, or Plaster Immobilization of Lower Extremities	+1
Bedridden for 3+ days, or major surgery in past 12 weeks involving general or regional anesthesia	+1
Deep Venous System Localized Tenderness	+1
Swelling of Entire Leg	+1
Calf Swelling ≥3 cm larger on other leg	+1
Pitting Edema only on symptomatic leg	+1
Collateral Superficial (Non-varicose) Veins	+1
Previously Documented DVT	+1
Alternative Diagnosis at least as likely as DVT	−2
Three-point Wells Score	
Low	<1
Intermediate	1–2
High	>2
Two-point Wells Score	
Unlikely	≤1
Likely	≥2
